# Urban Mobility Pattern Detection: Development of a Classification Algorithm Based on Machine Learning and GPS

**DOI:** 10.3390/s24123884

**Published:** 2024-06-15

**Authors:** Juan José Molina-Campoverde, Néstor Rivera-Campoverde, Paúl Andrés Molina Campoverde, Andrea Karina Bermeo Naula

**Affiliations:** Grupo de Investigación en Ingeniería del Transporte, Universidad Politécnica Salesiana, Cuenca 010105, Ecuador; nrivera@ups.edu.ec (N.R.-C.); pmolinac1@ups.edu.ec (P.A.M.C.); abermeon@ups.edu.ec (A.K.B.N.)

**Keywords:** transport mode, smartphone, GPS, pattern recognition, transportation mode detection, classification model, longitudinal dynamics

## Abstract

This study introduces an innovative algorithm for classifying transportation modes. It categorizes modes such as walking, biking, tram, bus, taxi, and private vehicles based on data collected through sensors embedded in smartphones. The data include date, time, latitude, longitude, altitude, and speed, gathered using a mobile application specifically designed for this project. These data were collected through the smartphone’s GPS to enhance the accuracy of the analysis. The stopping times of each transport mode, as well as the distance traveled and average speed, are analyzed to identify patterns and distinctive features. Conducted in Cuenca, Ecuador, the study aims to develop and validate an algorithm to enhance urban planning. It extracts significant features from mobility patterns, including speed, acceleration, and over-acceleration, and applies longitudinal dynamics to train the classification model. The classification algorithm relies on a decision tree model, achieving a high accuracy of 94.6% in validation and 94.9% in testing, demonstrating the effectiveness of the proposed approach. Additionally, the precision metric of 0.8938 signifies the model’s ability to make correct positive predictions, with nearly 90% of positive instances correctly identified. Furthermore, the recall metric at 0.83084 highlights the model’s capability to identify real positive instances within the dataset, capturing over 80% of positive instances. The calculated F1-score of 0.86117 indicates a harmonious balance between precision and recall, showcasing the models robust and well-rounded performance in classifying transport modes effectively. The study discusses the potential applications of this method in urban planning, transport management, public transport route optimization, and urban traffic monitoring. This research represents a preliminary stage in generating an origin–destination (OD) matrix to better understand how people move within the city.

## 1. Introduction

The technological development of telecommunication networks and the use of smartphones allow access to large volumes of data. Of the different groups of information that can be acquired through these devices, understanding how people can choose a means of transport to travel to their daily activities is an important area [1]. In recent years, the link between everyday means of transport and the costs they generate has led cities to promote mobility alternatives, such as cycling or walking to cover short distances, as well as developing more sustainable forms of mobility, such as encouraging the use of public transport by bus, train, etc. [2]. In an ever-growing urban environment, the decision on how to travel has become a key challenge, especially in demand management policies for passenger transport [3]. Determining the choice of transport mode provides essential data that can be used to improve urban planning and optimize the use of public transport, as well as to promote sustainable mobility in the urban area [4]. It is also important to consider that emissions generated by mobile sources powered by internal combustion engines cause high levels of air quality degradation in cities [5].

A recurrent issue in the literature concerns systems that collect accelerometer data, samples of which are used to train machine learning-based models capable of identifying the different modes of transport that a user can select, such as walking, cycling, driving a vehicle, taking a train, a bus, or using the underground, among others [6]. A prototype mobile application that operates in the background using geolocation and acceleration measurements to detect the user’s mode of transport represents a current problem due to changes in the accuracy and sensitivity of the systems used [7]. In one study, candidates placed the smartphone in their trouser pockets where samples were taken in different modes of transport, such as walking, standing still, running, cycling, car, bus, train, and metro [8]. Sensors are able to acquire details of variables from accelerometers, gyroscopes, and pressure sensors to predict the mode of transport used by the user based on previous measurements [9]. 

The main features used to categorize different transport modes include transport data, such as real-time geolocation on buses and rail lines, which are crucial for identifying urban mobility [10]. Patterns of average speed, average change in direction, and proximity to a bus location as well as stop times are determining factors for the identification of mobility characteristics [11]. Variables such as the average speed, stop times, and vehicle movement characteristics allow the identification of movement patterns that enable the determination of the means of transport in which a person travels using a mobile application [12].

Global positioning systems (GPSs) collect location, time, speed, and route data information to improve the accuracy of details of journeys [13]. GPS-based data collection has been shown to outperform traditional estimation methods, as well as to offer greater accuracy in terms of location and time at a lower cost [14]. Through the use of algorithms, key features of vehicle movement patterns are estimated, and a hierarchical decomposition is performed to detect these features by capturing signals from accelerometers [15].

In a complex urban environment such as Cuenca, Ecuador, located at 2500 m.a.s.l, where mobility is affected by congestion, emissions, and the need for sustainable alternatives, the authors of [16] suggest that innovative approaches are required to address these challenges. The study highlights a novel predictive model based on decision trees for the identification of a person’s mobility patterns, through the acquisition of geopositioning and movement data captured by a smartphone, capable of characterizing and identifying the mode of transport by which a person is moving.

In research on the characterization of human mobility patterns, significant progress has been made in the application of architectures based on deep learning. A prominent example is the implementation of the Generalized Linear Multi-Layer Perceptron for Transportation (GLMLP-TRANS), a system based on smartphone sensors that has proven to be effective in detecting the movement features of individual users [17]. Also, the use of techniques such as k-nearest neighbor and classification trees has shown high accuracy in characterizing transport modes, especially when considering frequency domain features [18].

In the field of transportation analysis, Transport Mode Detection (TMD) refers to the process of identifying and categorizing different modes of transportation based on data collected from various sources [19]. Important variables, such as acceleration and average speed, play significant roles in TMD, especially in considering the complexity of distinguishing between modes with similar movement profiles, such as buses, vehicles, and trains. In this context, smartphones and GPS recorders are invaluable tools for recording and analyzing data [20].

Sustainable urban development poses a significant challenge for cities worldwide, prompting the adoption of strategies like Transit-Oriented Development (TOD). TOD seeks to seamlessly integrate land use and transportation planning, aiming to foster more efficient mobility patterns while reducing reliance on automobiles. In this context, Wang et al. introduced the ANN-NPRT-HUB algorithm, specifically designed to analyze railway transportation networks within the framework of smart cities. Their pioneering work, illustrated through a case study in Chengdu, demonstrates the algorithm’s capacity to provide valuable insights into the classification and operation of railway transportation hubs within contemporary urban landscapes [21].

Amini Pishro et al., in their research, conducted a comprehensive investigation into Chengdu’s railway transportation stations. Their focus was on proposing the Node-Place-Ridership-Time (NPRT) model, which offers a nuanced understanding of passenger volume dynamics. Employing multiple linear regression (MLR), they meticulously examined the influence of node and place values on passenger volume. Additionally, the utilization of K-means and Cube methods allowed for the classification of stations based on NPRT model outcomes. Their findings suggest that the NPRT model presents a notable improvement over previous approaches in assessing the efficacy of railway transportation stations [22].

On the other hand, research conducted by Alotaibi et al. employed an ensemble learning approach to achieve robust model generalization. By acquiring data from smartphone accelerometers and gyroscopes, an effective approach to identifying driving patterns was achieved [20]. Furthermore, the study presented by Sadeghian et al. [23] highlights the importance of using machine learning architectures for transport mode detection by performing a systematic evaluation to select the most suitable algorithms.

The identification of driving influencing variables has been addressed by several authors. For instance, Ansari Lari et al. utilized a decision tree model to emphasize pivotal variables derived from the analysis of parameter identification data (PID) obtained through OBD II (on-board diagnostics) [24]. In a similar approach, Molina Campoverde has succeeded in classifying travel modes by attributes such as rates of change of speed and acceleration, obtaining accurate results with the application of decision tree models [25].

In a study focusing on characterizing the spread of COVID-19 from human mobility patterns and sociodemographic indicators, tree-based classifiers like Gradient Boosting were employed. The research compared the performance of different machine learning models, including Support Vector Machines and multinomial logit models. The findings revealed that tree-based classifiers, particularly Gradient Boosting, achieved the highest classification accuracy of 97.4% in predicting COVID-19 cases with an area under the curve (AUC) score of 0.987 [26].

Another study investigated leveraging travel behavior and personality profiles to nudge users toward more sustainable transportation choices. Using mobile sensing data, the researchers developed personalized interventions integrated into a route planning application. A pilot study involving 30 participants using the system for 6 weeks yielded positive evaluation results, indicating acceptance of the approach and instances of behavioral change [27].

In a separate study on mobility prediction and optimization of passenger traffic flows in smart city planning, various machine learning techniques were applied. By analyzing passenger traffic flows based on an Access, Egress, and Interchange (AEI) framework, mobility prediction models were developed. The research reported that the AEI framework achieved 91.17% prediction accuracy along with secure and lightweight encryption capabilities [28].

In addition, Ref. [29] presented a decision tree-based classification model using data collected from 16 candidates identified every 15 min on various modes of transport. This approach enabled effective identification of travel modes. Finally, Ref. [30] proposed an innovative method to estimate pollutant emissions based on specific driving characteristics, such as engine speed and throttle position, through the application of real driving emissions (RDE) obtained from OBD and GPS.

These studies collectively showcase the potential of machine learning and data-driven approaches in addressing urban transportation and sustainability challenges. Understanding the specific machine learning techniques employed, such as the use of tree-based classifiers and pre-processing steps applied to the data, provides valuable insights into the effectiveness of these methods.

Cuenca, Ecuador, like many other cities in Latin America, grapples with significant challenges related to transportation and sustainability. The rapid urbanization process has led to a sprawling cityscape, characterized by extensive infrastructure demands and high consumption of resources and energy [31]. This unchecked expansion, particularly towards the city’s peripheries, has resulted in an enlarged urban footprint and heightened emissions of pollutants. Addressing these pressing issues necessitates sustainable urban planning to regulate urban growth and enact essential measures. Ortiz et al. proposed a novel approach, integrating neighborhood sustainability assessment indicators with geographic information system (GIS) spatial analysis. This combined methodology aims to evaluate citywide sustainability and provide valuable insights to guide decision-making in Cuenca’s urban planning endeavors [32].

The present study proposes the development of an algorithm aimed at classifying mobility patterns in the city of Cuenca, Ecuador, using machine learning architectures. This algorithm aims to analyze and categorize the mobility behavior of the population based on data collected from mobile devices, such as speed, acceleration, position, and GPS. Furthermore, the study explores the application of machine learning techniques to characterize urban mobility patterns (walking, cycling, car, bus, taxi, and tram), providing insights that can be leveraged for urban planning and transport management in Cuenca. The present study was carried out with real data under normal traffic conditions for both pedestrians and means of transport without idealizing their values, and each variable was post-processed according to the physical and statistical phenomena that characterize them. The outcomes of this study are intended to serve as a foundation for generating an origin–destination matrix in a subsequent stage, which will further inform transportation planning and optimization efforts in the city.

The identification of areas with high levels of congestion or low accessibility could provide valuable insights for informing the planning of new transportation infrastructures or optimizing public transportation routes. Highlighting the promotion of more sustainable mobility options, such as walking, cycling, or using public transport, could further contribute to addressing urban transportation challenges and fostering environmentally friendly transport practices.

Accurate classification of transportation modes offers a valuable tool for urban planners and transportation managers. The data collected and analyzed through the proposed algorithm could inform policy decisions related to new transportation infrastructure, such as the placement of bicycle lanes or public transit stops. This information could support sustainable mobility initiatives by providing insights into citizens’ preferences for eco-friendly modes of transport. City planners could use the classification model to understand commuting behaviors and target investments toward improving public transit connectivity and frequency in high-demand areas. Transportation agencies could also leverage the model’s outputs to identify neighborhoods where bike-sharing programs or pedestrian-friendly designs would be most effective in encouraging the adoption of sustainable mobility options.

This article is structured as follows: Section 2 describes the data acquisition process, detailing how the mobility data were collected using a mobile application and the specific variables captured, in addition to the methodology for developing the classification algorithm, including the selection of relevant features and the implementation of machine learning techniques. In Section 3, the results obtained from evaluating the performance of the proposed algorithm are presented. A comparison with existing methodologies is conducted in Section 4. In this section, the implications of the findings for urban mobility research are discussed, and potential future directions are explored. Finally, conclusions and consideration of future work are presented in Section 5.

## 2. Materials and Methods

### 2.1. Data Collection and Analysis

**Figure 1:**
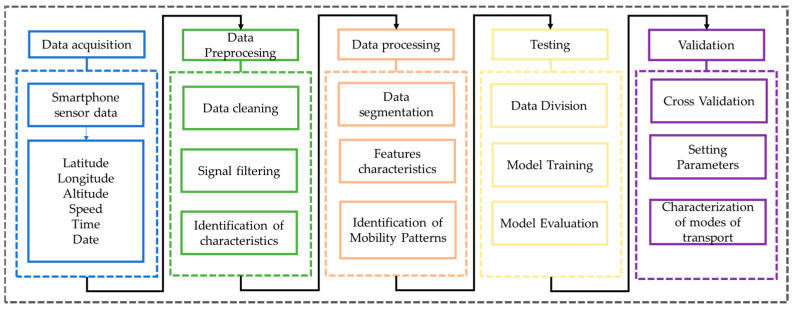
Methodology and proposed procedure.

Figure 1 illustrates the methodological framework of this study, which starts by gathering data from various smartphones. Subsequently, this information undergoes a cleaning and filtering process to eliminate outliers and signal noise. Then, the data are segmented to extract model features. Moreover, informed consent is obtained from each participant for data acquisition solely for research purposes, thus ensuring data integrity and adherence to ethical principles in research conduct.

**Figure 2:**
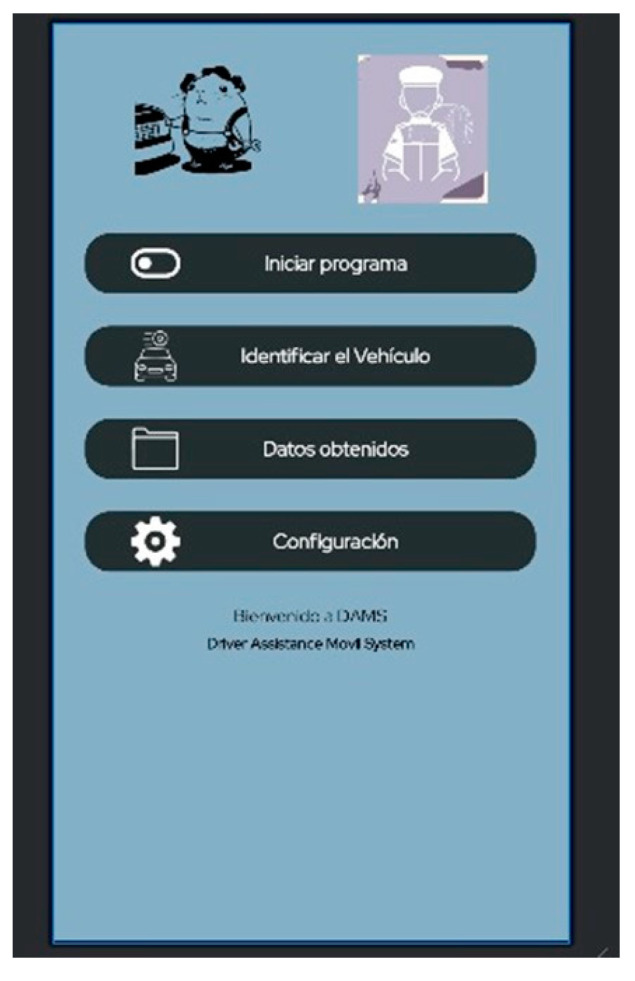
Mobile app developed by the research group.

The application developed for the acquisition of data from smartphones allows variables such as speed, position, time, latitude, and longitude to be obtained. In addition, in the first phase of data acquisition, monitoring was carried out employing a mobile application that constantly records data through an application designed specifically for this purpose. Once the user downloads the application and accepts the location permissions, the activities are recorded in real-time. In previous research, low-cost inertial sensors embedded in recent-model smartphones have been used for data collection. Previous studies have shown that the inertial sensors embedded in smartphones are capable of providing angular displacement measurements with a maximum uncertainty of 0.3°, indicating a very low measurement uncertainty. This supports the use of these sensors in this project for the measurement of mobility patterns [33,34]. In Figure 2, the mobile application developed by the research group is shown. On the left-hand side of the screen, a menu is displayed with options such as “Start program”, “Identify means of transportation”, “Data obtained”, and “Settings”, each accompanied by an icon. Furthermore, this application allows for the collection of data from various modes of mobility, with the capability to export them as text files.

Apolo et al. conducted a comparison between the data collected through a mobile application specifically developed for the project and those provided by a Freematics data logger to validate the obtained results. Additionally, the study employed cross-validation for data acquisition. The authors calculated correlation and determination coefficients between the values recorded by both devices. The correlation coefficient had a value of 0.99847, while the determination coefficient R^2^ was 0.99695. These findings signify a high correspondence between the measurements taken by the mobile application and the data logger, thus affirming the validity and reliability of the data obtained through the proposed solution [35].

Throughout November 2023, the variables captured by the smartphone’s accelerometer and GPS were collected at a sampling frequency of 1 s. These measurements were taken at various time slots and days of the week while participants carried the devices in their trouser pockets. The acquired data formed a matrix of 953,819 × 6 elements, as detailed in Table 1. The sampled variables are shown below.

The distribution of different modes of transportation in the 953,819 × 6 matrix was monitored and recorded during the data collection process. The sampling was designed to capture a representative proportion of each mode: Bicycle at 9.81%, Bus at 28.01%, Walking at 5.17%, Tram at 7.77%, Taxi at 30.55%, and Vehicle at 18.69%. This strategy ensured that the dataset accurately reflected the real-world usage of various transportation modes within the study area. A randomized sampling process was employed to guarantee an unbiased and representative selection of data, with a random approach used for participant selection and time interval assignment for data capture. Rigorous measures were taken to minimize any possible bias, ensuring that the sampled percentages accurately represented the actual distribution of transport modes in the study area.

In the study, we meticulously considered potential sources of bias in both the dataset and the model. To address these concerns, we implemented several strategies aimed at mitigating biases. Firstly, we employed a diverse recruitment strategy to ensure representation across various demographic groups, promoting the application through multiple channels and targeting a wide range of potential users. Additionally, we carefully monitored participant demographics throughout the data collection process to identify any potential biases and adjusted our sampling methods accordingly. We also utilized statistical techniques such as stratification and weighting to account for any observed imbalances in the sample. By employing these rigorous methodologies, we aimed to enhance the robustness and generalizability of our findings, thus minimizing the impact of potential biases on the study results.

**Figure 3:**
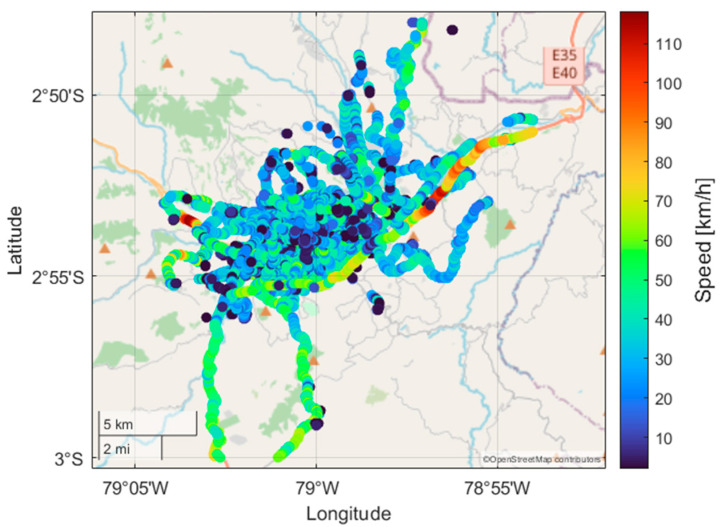
Distribution of random speeds for transportation modes.

Subsequently, the analysis and computational processing of the data were conducted using MATLAB R2023a. In this phase, the data underwent meticulous preprocessing and cleaning procedures within MATLAB to eliminate noise, ensure data quality, and enhance accuracy for subsequent analysis. The data preprocessing and cleaning steps were crucial to maintaining data integrity and reliability. This study was conducted in the city of Cuenca, Ecuador, situated at an average altitude of 2500 m above sea level, with stable environmental conditions and an average temperature of 17 °C. Cuenca has a population of 596,101 inhabitants according to the 2022 census [36]. Figure 3 illustrates the speed profiles of the samples collected by each of the mobile devices monitored.

**Figure 4:**
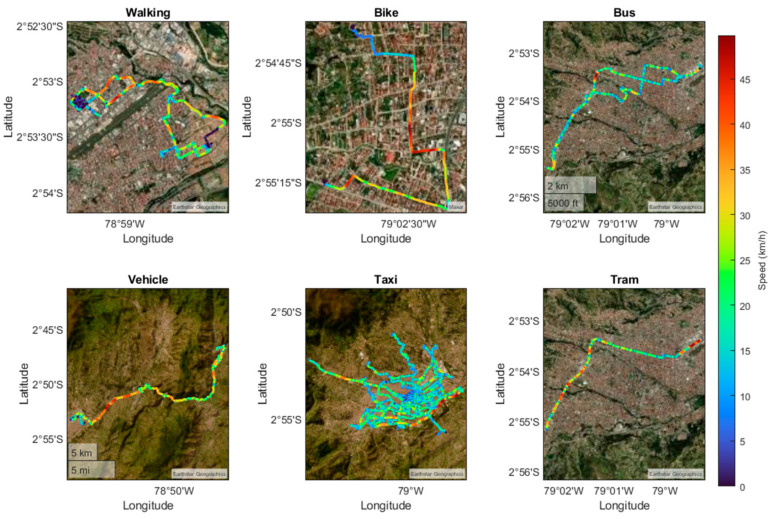
Routes traveled by each mode of transport.

Figure 4 shows different routes taken by various means of transport, such as walking, cycling, taking a taxi, and using a private vehicle. These routes are analyzed with the purpose of extracting the characteristics obtained under normal conditions, including the speed during the journey in each means of transport. The geographical coordinates of each route can also be identified, indicating the latitude and longitude of the start and end points of each route.

### 2.2. Segmentation and Classification of Activities

**Figure 5:**
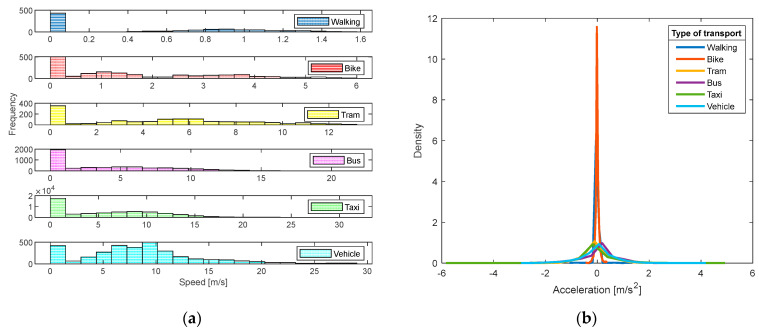
Speed (**a**) and acceleration (**b**) profiles.

To identify each of the characteristics of each means of transport, the recorded activity was studied separately. It is important to mention that each means of transport generates different velocity and acceleration profiles, which is why it is necessary to carry out an exploratory analysis. Speed histograms are used to analyze the speed recorded by each mode of transport, as shown in Figure 5, while the acceleration profile provides information on the rate of change of speed over time. For example, the profile for the bicycle shows a low acceleration range, but with a high density in that area, while that for the private vehicle exhibits a much wider acceleration range, due to the different driving patterns identified by each driver.

**Figure 6:**
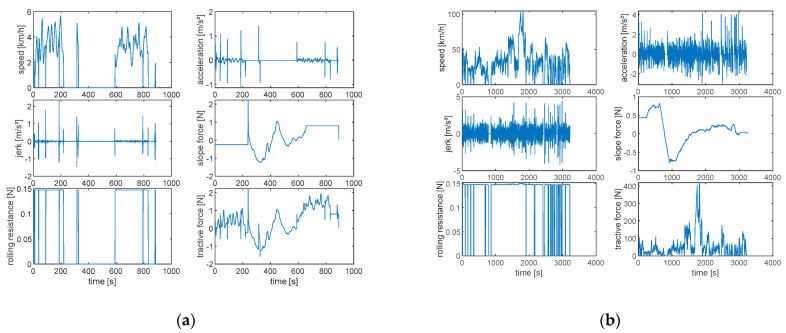
Application of longitudinal dynamics: walking (**a**) and vehicle (**b**).

To analyze mobility characteristics in-depth, certain variables, such as acceleration, jerk (rate of change of acceleration over time), and road slope, a parameter necessary for the estimation of gravitational force, are estimated. Additionally, from the recorded data, it is possible to calculate the aerodynamic drag force and the rolling resistance, as shown in Figure 6. With these estimates, all the resistances that oppose the movement of the vehicle are determined. Finally, the average speed of each path is calculated. These analyses provide an understanding of the behavior of the means of transport under various conditions, which is crucial for assessing its performance and efficiency.

**Figure 7:**
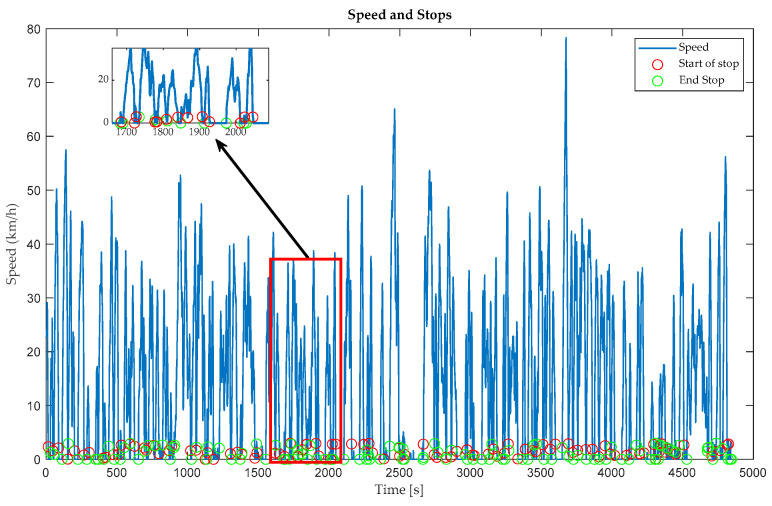
Detection of downtimes.

Using Figure 7, characteristics such as the average speed, distance traveled, and stopping time can be analyzed for each mode of transport. This graph also allows us to examine the movement patterns of each of these modes of transport. The evolution of distance over time is considered and significant variations in different areas or conditions are looked for. In addition, the stopping times are investigated, which may show moments of inactivity or rest during each journey.

The three-dimensional scatter plot, on the other hand, shows how these modes of transport are grouped and differentiated according to kinematic variables, such as acceleration and momentum. These visualizations suggest that data collected through mobile device sensors has the potential to effectively distinguish between users’ forms of mobility.

Time series represent the variation in an indicator over time, which is possibly related to the detection of periods of inactivity or downtime during user mobility. These graphs allow visual identification of times when there are dips, plateaus, or sharp changes in the monitored parameter, which could signify transitions between periods of activity and periods of pause or stop.

The three-dimensional scatter plot shows the relationship between kinematic variables, such as acceleration and momentum, and the mode of transport. This multivariate representation could provide valuable information to distinguish between segments of activity and inactivity within mobility patterns. Certain patterns or clusters in this visualization could be associated with stopping or waiting times for different modes of transport. The integrated analysis of these graphs could contribute to the accurate identification of idle times or downtimes in users’ journeys.

**Figure 8:**
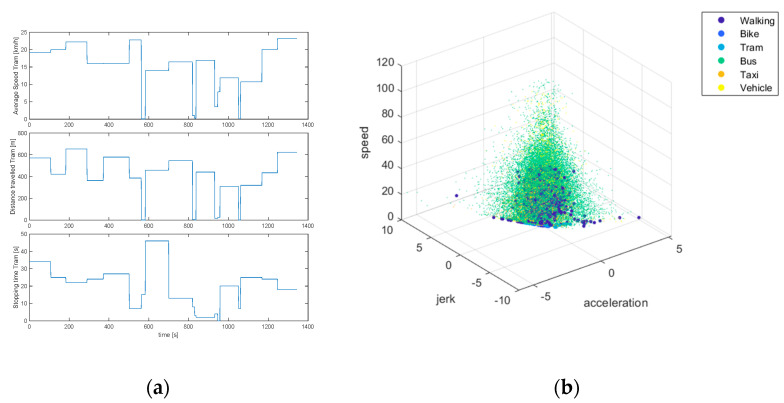
Characteristics of transport modes. (**a**) Average speed, distance, and travel time for tram; (**b)** 3D scatter plot of speed, acceleration, and jerk for various modes of transport.

In Figure 8, some of the most important characteristics of different modes of transport, such as walking, bicycle, tram, bus, taxi, and private vehicles, can be visualized in three-dimensional form. Additionally, this graph can be used to examine speed, acceleration, and jerk, as well as to allow the identification of patterns and ranges of movement between modes of transport. This type of three-dimensional graphical representation facilitates the identification of patterns and ranges of movement specific to each mode of transport. For example, it can be observed that private vehicles tend to have higher values of speed and acceleration, while modes such as walking and tram present smoother and more uniform profiles.

The importance of each analysis and visualization lies in their ability to provide insights into the intricate dynamics of mobility patterns and the distinctive characteristics of different transport modes. For example, analysis of speed variations can reveal the efficiency and accessibility of different transport options, while visualization of route density highlights areas of high demand or congestion. These analyses contribute to a comprehensive understanding of urban mobility, assisting in the optimization of transport systems and the development of sustainable transport policies. In addition, the research in this study has direct implications for addressing real-world transport challenges and advancing knowledge of urban mobility. By providing practical information derived from empirical data, this research can enable policymakers and urban planners to make informed decisions on infrastructure development, traffic management strategies, and initiatives that promote sustainable transport alternatives.

### 2.3. Travel Mode Detection

This section presents an analysis of longitudinal dynamics to examine the main forces opposing movement in each mode of transport, with the purpose of determining their patterns and characteristics in relation to physical variables.

A longitudinal dynamics analysis is carried out to examine the main forces opposing the movement of each means of transport, with the purpose of determining their patterns and characteristics in relation to physical variables.

A unit value for mass m=1  kg, aerodynamic adhesion coefficient $C_{X}=1$, frontal area $A_{f}=1$ m^2^, and density ρ=1 kg/m^3^ are considered, due to the variety of means of transport under study. In this analysis, a normalization approach was adopted by using reference values equal to 1 for certain coefficients, which allows greater flexibility in considering a variety of media with the advantage of simplifying the calculations.

The driving force $F_{T}$ is related to all the opposing forces that each medium must overcome in order to move, including the aerodynamic resistance $F_{a}$, rolling resistance $F_{res}$, longitudinal acceleration $a_{x}$, braking force $F_{brk}$, and slope resistance $F_{slope}$ [37].

(1) $$a_{x}=F_{T}-F_{res}-F_{slope}-F_{brk}$$

where the longitudinal acceleration $a_{x}$ is calculated using the GPS velocity as a reference:

(2) $$a_{x}i=\frac{V_{GPS\;i+1}-V_{GPS\;i}}{t_{i+1}-t_{i}}$$

The rolling resistance is represented as $f_{r}=f+f_{0}{\left(\frac{V}{100}\right)}^{2.5}$, where the static and dynamic rolling coefficients are f=0.015 and $f_{0}=0.01$, respectively. The slope resistance is obtained from [38]:

(3) $$F_{res}=mg\left(f+f_{0}{V_{GPS\;i}}^{2.5}\right)+\frac{1}{2}\rho {{C_{X}A_{f}V}_{GPS\;i}}^{2}$$

The slope force is defined as the resistance experienced when ascending a slope.

(4) $$F_{slope}=mg\sin\left(\frac{{Alt}_{\;i+1}-{Alt}_{\;i}}{S_{i+1}-S_{i}}\right)$$

Finally, in Table 2, the traction force is presented, which is determined using the data obtained during the smartphone’s travel.

(5) $$F_{T}-F_{brk}=ma_{x}+F_{res}+F_{slope}$$

Table 3 presents a descriptive statistical analysis examining the speeds and accelerations of various vehicle categories, such as pedestrians, bicycles, trams, buses, taxis, and cars. For each mode of transport, various metrics are calculated, including the mean, maximum, and minimum speed, standard deviation, root mean square (RMS) of speed, as well as acceleration metrics, such as maximum, minimum, and mean. Additionally, the number of directional changes in acceleration is recorded and the stopping time is determined based on a predefined speed threshold. Table 3 presents a summary that provides comparative information on the behavior and characteristics of each vehicle type in terms of its movement, which is relevant for mobility and traffic analysis.

## 3. Results

### 3.1. Characterization of Transport Modes Using the Decision Tree

Uniform information was used as input for the decision tree across all modes of transportation. The input variables considered for each mode included speed, acceleration, jerk, gravitational forces, traction forces, average speed, downtimes, and distance traveled. The output corresponds to the label of each respective mode of transportation. These inputs represent specific variables for each mode, facilitating the classification and prediction of different transport modes.

**Figure 9:**
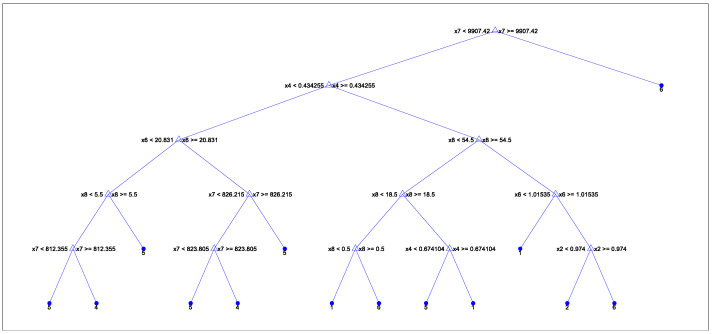
Decision tree.

Trees store the bag of 100 trained regression trees in a 100-by-1 cell array. However, for visualization purposes, the tree shown in Figure 9 has been simplified and condensed to only 15 splits to make it more readable and understandable.

A dataset consisting of 64,831 observations was used to train a decision tree model. The training process included validation through a 5-fold cross-validation. Eight variables were used as predictors to predict the response variable ‘Label’, which has six different classes. The model was fitted using the training data and its performance was evaluated through cross-validation.

**Figure 10:**
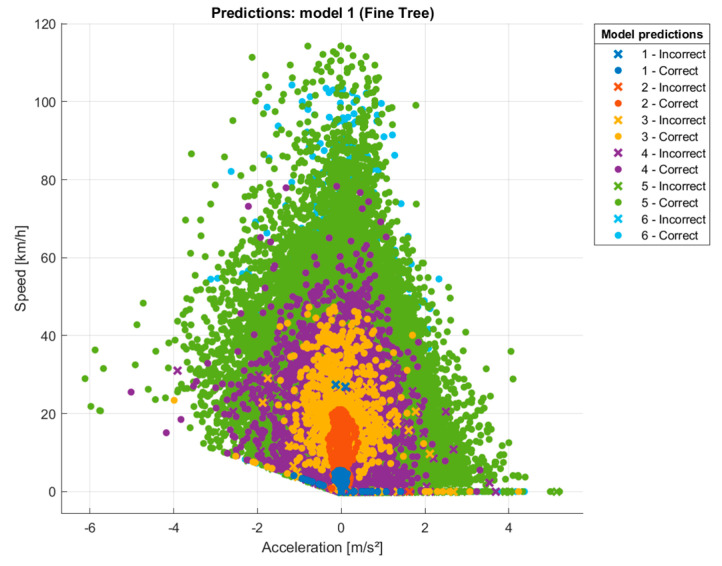
Speed vs. acceleration prediction model.

After extracting the various metrics and highlighting that acceleration is a highly relevant predictor, the next step is to train a model using a decision tree, as shown in Figure 10. The transport modes are represented by numbers ranging from 1 to 6 (1, 2, 3, 4, 5, 6) and whose labels correspond to the following transport modes: Transport Mode 1: Walking, Transport Mode 2: Bike, Transport Mode 3: Tram, Transport Mode 4: Bus, Transport Mode 5: Taxi, Transport Mode 6: Vehicle. In the validation stage, this decision tree model presents an accuracy of 94.6%, indicating a highly satisfactory performance in classifying the different transport modes based on the predictor features.

Figure 10 shows a decision tree predictive model that analyzes the relationship between speed (km/h) on the y-axis and acceleration (m/s²) on the x-axis for different modes of transport, identified by numerical codes from 1 to 6. Each point in the diagram corresponds to a model prediction, with colors indicating whether the estimate was correct (green) or incorrect (other colors). Crosses represent incorrect predictions, while circles represent correct predictions. The colors range from blue, green, and purple to orange and other shades, differentiating the categories.

This legend shows whether the prediction was correct or incorrect for six different categories, with each category represented by a number from 1 to 6 and with separate entries for ‘Correct’ and ‘Incorrect’.

The analysis of this type of graphical representation allows the identification of distinct performance patterns among the different modes of transport studied. The model demonstrates higher predictive accuracy for some modes, such as walking and cycling, compared to others, like taxis and private vehicles. The data points are dispersed across the graph, with a concentration of points around lower acceleration values and higher speed values. There is a noticeable trend where the density of points is higher in the center of the graph, suggesting that the model’s predictions may be more accurate in this region.

This information is valuable for understanding the specific dynamics of each transport type, which can lead to improvements in urban mobility modeling and representation.

### 3.2. Model Validation through Testing

**Figure 11:**
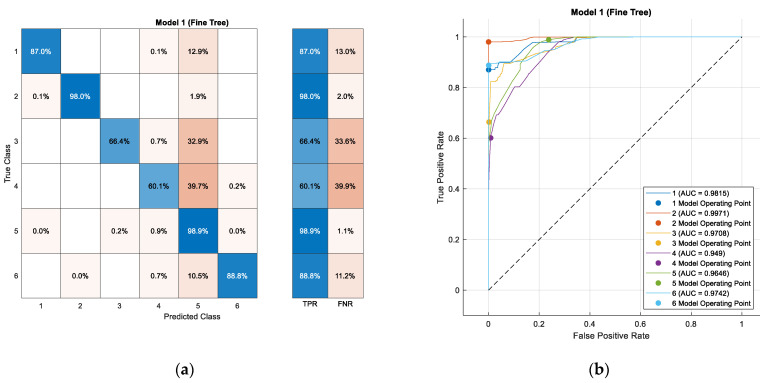
Validation confusion matrix (**a**) and classification tree validation ROC (**b**).

A prediction matrix is visualized in Figure 11a. Each row of the matrix represents the instances in a real class (true classes), while each column represents the instances in a predicted class (predicted classes). A total of 94.6% of the predictions made by the model during the validation stage were correct—a high validation accuracy indicates that the model generalizes the data exhibited during training well.

Table 4 shows the confusion matrix of the data validation process. Here, the total number of observations is displayed and an accuracy of 94.6% is obtained, calculated from the number of correct predictions concerning the total number of observations in the model.

In the analysis of the confusion matrix, areas were identified where the transportation mode classification model may include classification error. It was noted that the model tends to conflate the classes associated with the “Tram” (Transport Mode 3) and “Bus” (Transport Mode 4) modes, indicating similarities in the data that could result in misclassifications between these two specific modes. These findings provide a deeper insight into potential sources of error in the model and underscore the importance of addressing these areas to enhance accuracy in the classification of transportation modes.

The precision is an essential metric that reveals the proportion of correct predictions made by the model, with the value obtained of 0.8938 indicating that almost 90% of instances predicted as positive are truly positive. This high accuracy in positive predictions is crucial for confidence in the model.

(6) $${Precision}_{i}=\frac{CM(i,i)}{\sum_{j}CM(j,i)}=0.894$$

Regarding recall, measured at 0.83084, this is another indicator showcasing the model’s ability to correctly identify real positive instances within the dataset. With a value of 83.084%, it suggests that the model can capture over 80% of all positive instances present in the data, demonstrating notable effectiveness in finding positive instances.

(7) $${Recall}_{i}=\frac{CM(i,i)}{\sum_{j}CM(i,j)}=0.831$$

The F1-score represents the harmonic mean of precision and recall, indicating a good balance between these two metrics—in this case, a value of 0.86117 was obtained. This suggests that the model not only boasts high precision and good recall viewed individually but that, using a composite measure combining these metrics, it exhibits solid and balanced overall performance.

(8) $$F1-Score=\frac{2\cdot Precision\cdot Recall}{Precision+Recall}=0.861$$

A prediction matrix is shown in Figure 11a. Each row of the matrix represents instances of the true classes, while each column represents instances of the predicted classes. In this case, the model achieves an accuracy of 83.2%, calculated by summing the correct predictions and dividing by the total number of predictions. This overall accuracy indicator complements the details provided in the confusion matrices, which allow the performance of each class to be analyzed. The prediction matrix and confusion matrices show true positive rates (TPR) and false negative rates (FNR) for each class, providing further insight into the model’s performance in accurately identifying each class and the instances where it failed to do so.

In the analysis of individual classes, it is observed that Walking has a true positive rate of 87.0% with very little confusion with other classes. For the Bike scenario, a correct classification rate of 98% is achieved, while Walking and Tram exhibit a true positive rate of 66.4%. Vehicle exhibits a false positive rate of 32.9%, while Bus achieves a correct classification rate of 60.1%. Taxi stands out with an accuracy of 98.9%, and lastly, Vehicle shows an 88.8% correct classification rate.

The receiver operating characteristic (ROC) curve shown in Figure 11b, which is commonly used to evaluate the performance of a classifier system, shows the true positive rate on the y-axis, while the false positive rate can be visualized on the x-axis, both with values between 0 and 1.

In the ROC analysis for the transport modes Walking, Bike, Tram, Bus, Taxi, and Vehicle, it is observed that each ROC curve represents the performance of a binary classifier model to distinguish between two classes. Each curve is associated with a specific mode of transport and shows the true positive rate as a function of the false positive rate. The area under the curve (AUC) values provide a measure of the discrimination ability of each model, where it is highlighted that the model associated with Bike has the best performance with an AUC of 0.9971, closely followed by Walking with an AUC of 0.9815. This indicates that these models have a high accuracy in classifying the corresponding transport modes, while the model associated with Vehicle shows the lowest AUC of 0.9472, which still suggests an acceptable classification performance, as visualized in Figure 11.

### 3.3. Model Evaluation and Validation through Testing with Random Datasets

In the evaluation phase, the model has an accuracy of 94.9%, which demonstrates an excellent generalization capacity of the model to correctly classify the modes of transport on data independent of those used during training. This high level of accuracy in the test stage reinforces the robustness and reliability of the model developed for the identification of the different modes of transport based on the characteristics.

The test confusion matrix (Table 5) illustrates both correct and incorrect classifications made by the model on the test dataset. It is evident that the model encounters challenges in accurately distinguishing between the classes corresponding to the “Bus” and “Taxi” transport modes (Transport Mode 4 and Transport Mode 5), likely due to similarities in the data. This observation leads to a higher confusion rate between these specific classes. These findings underscore the significance of addressing areas where the model encounters difficulty, aiming to enhance its precision in classifying the identified transport modes.

**Figure 12:**
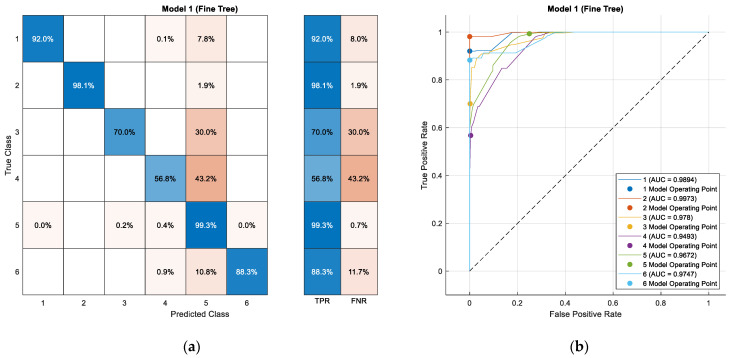
Test confusion matrix (**a**) and classification tree test ROC (**b**).

Similar to the previous case, 94.9% of the predictions made by the model during the testing stage were correct. This means that the model was correct in classifying 94.9% of the samples in an independent dataset that were not used during training and validation. This is verified by Figure 12b using the ROC.

The prediction matrix and the confusion matrices show true positive rates (TPR) and false negative rates (FNR) for each class, providing further insight into the model’s performance in accurately identifying each class and the instances where it failed to do so. During testing, the model achieved an accuracy of 84.8%, demonstrating consistent performance across different datasets.

### 3.4. Characterization of Transport Modes Using the Decision Tree

**Figure 13:**
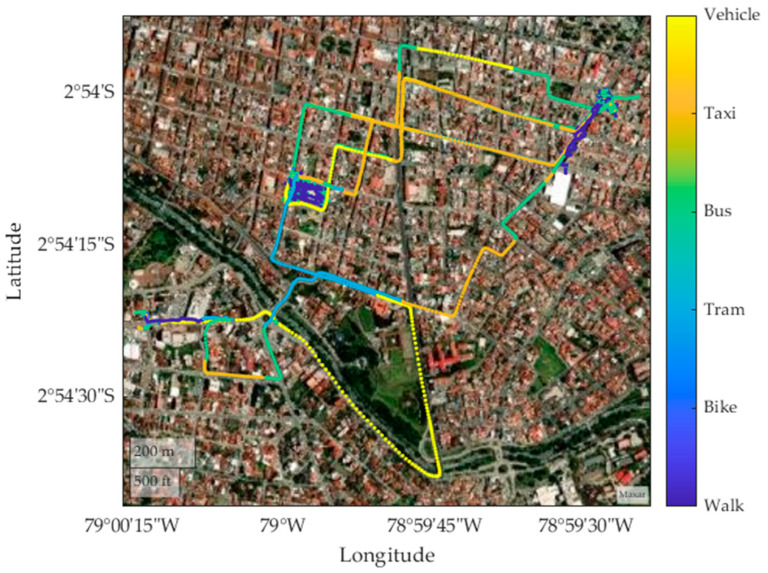
Characterization of transport modes by color random samples 1.

The analysis presented in Figure 13 shows a classification of various modes of transport present in each geographical area. By graphically representing the trajectories corresponding to each mode, including vehicles, taxis, buses, trams, bicycles, and pedestrians, a clear visualization of the spatial distribution and mobility patterns in the study area is achieved. This approach allows for a comprehensive understanding of the dynamics of the transport system, identifying areas of greater or lesser activity, and providing valuable information to support urban mobility planning and management in the context analyzed.

**Figure 14:**
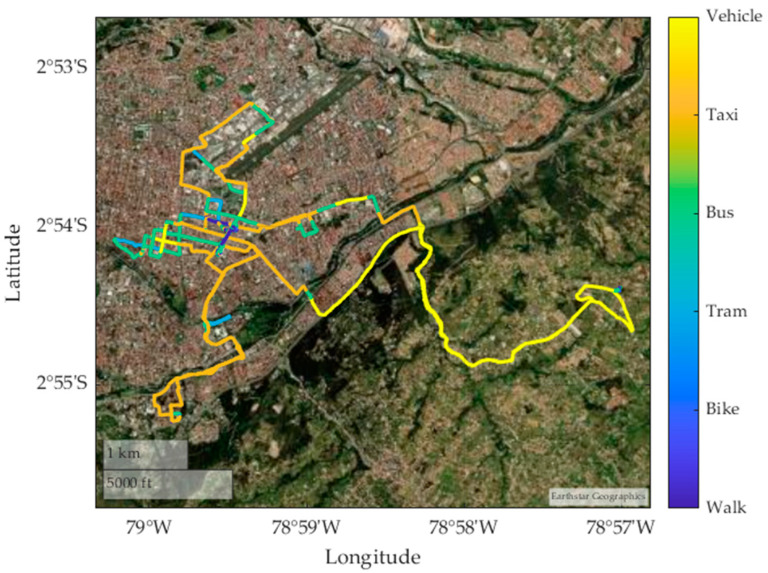
Characterization of transport modes by color random samples 2.

Figure 14 shows a graphical representation of the different modes of transport classified from random data. The colors used in the trajectories indicate the different modes of travel identified by the model, including car (yellow), taxi (orange), bus (blue), tram (green), bicycle (purple), and walking (light blue). This visualization allows the accuracy of the model in classifying user mobility patterns to be assessed, determining its generalizability to correctly predict the mode of transport used and identifying potential areas for improvement or transport patterns that require further adjustments to the model.

## 4. Discussion

In this study, we have presented a classification model based on decision trees to determine transport modes using smartphone data acquisition with an accuracy of 94.6% in the validation and 94.9% in the test. Compared to previous studies, our model significantly outperforms the accuracy of other approaches, such as the algorithm implemented by [39], which achieved an accuracy of 82% in Zurich, Switzerland. Ref. [40] demonstrated an accuracy of 97.02% when combining temporal and frequency features. In addition, previous researchers [41] achieved 93.5% accuracy in transport mode classification by extracting transport network features. Other works also support the effectiveness of neural network classifiers for transportation mode detection (TMD), with 94.6% and 94.5% accuracy and recall, respectively [42,43].

The study *‘Topic Classification from Text Using Decision Tree, K-NN, and Multinomial Naïve Bayes’* investigated the performance of three different classifiers in a topic classification task with six classes. The obtained results include a precision of 0.794, recall of 0.788, and an F1-score of 0.790. In contrast, the results of the present study demonstrate a significantly higher precision of 0.8938 and an F1-score of 0.86117. This suggests a substantial improvement in classification capability achieved through our approach, highlighting the effectiveness of the techniques employed in detecting patterns of transportation mode mobility compared to topic classification in text [44].

In a study of random forests and decision trees, a comparison between Random Forest and J48 for classifying diverse datasets highlighted the performance of different classification models across various dataset sizes. Specifically, Random Forest showed superior performance with larger datasets, while J48 was more effective with smaller datasets. For instance, the precision increased from 0.667 to 0.962, the F1-score from 0.674 to 0.961, and recall from 0.692 to 0.961 for the Random Forest classifier as the number of instances increased. In contrast, the precision, F1-score, and recall achieved in the present study, with a precision of 0.8938, recall of 0.83084, and an F1-score of 0.86117, reflect the efficacy of the approach in classifying datasets of various sizes [45].

As we look forward to future applications, our model aims to tackle the challenge of increasing traffic congestion during peak hours in urban areas. By utilizing the precise classification of transport modes offered by our model, city planners can pinpoint specific corridors or intersections with the highest congestion levels. Equipped with this information, authorities can implement targeted measures, such as optimizing traffic signal timings, designating bus lanes, or introducing congestion pricing schemes, to alleviate traffic bottlenecks and enhance overall traffic flow [46].

Chavhan et al. proposed a traffic management system based on predictive information, utilizing static and mobile agents that collect and share traffic flow data, such as speed and density, to predict traffic patterns and optimize routes in real time, thereby reducing congestion and improving traffic flow. On the other hand, our study focuses on the accurate classification of transport modes, providing essential information for urban planning. This allows for the identification of congestion areas and infrastructure needs, as well as the development of specific strategies to alleviate congestion, such as promoting carpooling and enhancing public transportation in high car usage areas during peak hours [47]. 

In the study *‘Efficient Detection of Botnet Traffic by Feature Selection and Decision Trees’*, the emphasis is placed on improving the classification of botnet traffic through feature selection, employing techniques such as information gain and Gini importance. This methodology yielded an average F1-score of 85% with decision trees. In contrast, our research achieved a slightly higher F1-score of 86.11% [48].

During the development and evaluation of the mobility pattern classification model, several limitations and challenges were encountered. One of the primary challenges was data imbalance. This occurs when certain transportation modes are underrepresented in the dataset, leading to biased model predictions. Feature selection also posed a significant challenge. With numerous potential features derived from the data, it was crucial to identify the most relevant ones to enhance the model’s performance and reduce computational complexity. Overfitting, where the model performs well on training data but poorly on unseen data, was another critical issue. This was mitigated by implementing several strategies. Cross-validation techniques, specifically, k-fold cross-validation, were used to ensure that the model generalized well to new data.

To enhance the model’s predictive accuracy and applicability in urban mobility contexts in Cuenca, Ecuador, it is advisable to discuss potential extensions or improvements. In our current model, decision trees were employed alongside features like speed characteristics and longitudinal dynamics. Additional data, such as distance traveled, average speed, and stop times, were collected through smartphone data acquisition to characterize different modes of transport. Future enhancements could entail the integration of more diverse data sources, including weather conditions, real-time traffic updates, and public transportation schedules. Moreover, experimenting with advanced machine learning algorithms, such as deep neural networks or ensemble methods, may further enhance the model’s performance, offering urban planners more precise and actionable insights [49].

## 5. Conclusions

This study presents a decision tree-based transport mode classification algorithm using data collected from sensors embedded in smartphones through a specially developed application. The data include variables such as date, time, latitude, longitude, altitude, and speed. Apolo et al., who worked on the application for this project, found a high correspondence between the measurements taken by the mobile application and a data logger, with a correlation coefficient of 0.99847 and a determination coefficient R^2^ of 0.99695. These results confirm the validity and reliability of the data obtained through these technologies.

Significant features of mobility patterns, such as the maximum, minimum, mean, and RMS values of variables like velocity, acceleration, and over-acceleration, were extracted. Additionally, longitudinal dynamics, such as the forces ${F_{r},\;F}_{res},\;F_{slope},\;and\;F_{i}$, were applied to feed the classification model. Stopping times for each transport mode, distance traveled, and average speed were analyzed to identify patterns and distinctive features. The test results showed an accuracy of 94.6% in validation and 94.9% in testing, demonstrating the effectiveness of the proposed algorithm in accurately classifying transport modes.

Additionally, the precision metric of 0.8938 signifies the model’s ability to make correct positive predictions, with nearly 90% of positive instances correctly identified. Furthermore, the recall metric at 0.83084 highlights the model’s capability to identify real positive instances within the dataset, capturing over 80% of positive instances. The calculated F1-score of 0.86117 indicates a harmonious balance between precision and recall, showcasing the model’s robust and well-rounded performance in classifying transport modes effectively.

The outcomes of this study are intended to serve as a foundation for generating an origin–destination (OD) matrix in a subsequent stage, which will further inform transportation planning and optimization efforts in the city of Cuenca, Ecuador. Cuenca is a relatively small city with a population of 596,101 according to the 2022 census, and it does not have a metro system, which may limit the diversity of mobility patterns observed. Additionally, the city’s high altitude of 2500 m above sea level and irregular geography could introduce unique challenges in terms of transportation infrastructure and user behavior that may not be directly applicable to other urban contexts.

Our discussion highlights the potential limitations associated with the specific characteristics of Cuenca, which may affect the generalizability of our findings to other urban settings. While focusing on Cuenca allows for an in-depth understanding of traffic congestion dynamics in this city, unique factors, such as the population density, infrastructure layout, and cultural influences, may limit the direct applicability of our results to larger or differently structured cities. Future research should address these variations to enhance the transferability of our findings across diverse urban environments.

Additionally, to improve the model’s predictive accuracy and utility in urban mobility contexts, incorporating more features and exploring alternative machine learning algorithms, such as deep neural networks or ensemble methods, is recommended. Future research could also consider integrating the data collected via smartphones with other sources of information, such as global positioning systems (GPSs), public transport card records, or real-time traffic data. This combination of diverse sources could strengthen both the accuracy and robustness of the mode classification model.

Based on the results obtained, developing mobile applications or software solutions that allow end-users to monitor and understand their mobility patterns could be advanced to promote more efficiency and sustainability. These efforts will support more effective urban planning and management in Cuenca and similar cities.

## Figures and Tables

**Table 1:** Data registered by the smartphone.

Characteristics	Units
Date	yyyy-MM-dd
Time	hh:mm:ss
Latitude	degrees (°) S
Longitude	degrees (°) W
Altitude	m.a.s.l
Speed	m/s

**Table 2:** The results obtained after applying longitudinal dynamics to the collected data are shown below.

Speed [km/h]	Acceleration [m/s^2^]	Jerk [m/s^3^]	Slope Force [N]	Aerodynamic Force [N]	Rolling Resistance [N]	Tractive Force [N]	Average Speed [m/s]	Mode Classification
4.03	0.68	−0.70	1.83	0.63	0.15	3.28	2	1
6.48	−0.01	−0.22	1.84	1.62	0.15	3.59	2	1
6.43	−0.23	0.27	1.85	1.59	0.15	3.36	2	1
5.59	0.03	0.13	1.85	1.20	0.15	3.24	2	1
15.54	0.08	0.01	0.25	9.32	0.15	9.80	6	2
15.82	0.08	−0.03	0.26	9.65	0.15	10.14	6	2
16.12	0.05	0.02	0.26	10.02	0.15	10.48	6	2
16.31	0.08	0.03	0.26	10.26	0.15	10.74	6	2
32.53	0.45	−0.13	0.02	40.83	0.15	41.45	16	3
34.15	0.32	−0.09	0.02	44.98	0.15	45.47	16	3
35.28	0.22	−0.12	0.02	48.03	0.15	48.42	16	3
36.08	0.10	0.30	0.02	50.23	0.15	50.50	16	3
35.28	0.64	0.14	0.02	48.02	0.15	48.83	14	4
37.58	0.78	−0.69	0.02	54.50	0.15	55.44	14	4
40.39	0.09	0.68	0.02	62.94	0.15	63.20	14	4
40.72	0.77	−0.31	0.02	63.96	0.15	64.90	14	4
37.98	−0.09	0.14	−0.05	55.65	0.15	55.66	21	5
37.66	0.05	0.06	−0.05	54.71	0.15	54.85	21	5
37.84	0.11	0.24	−0.05	55.23	0.15	55.44	21	5
38.23	0.35	0.36	−0.05	56.39	0.15	56.84	21	5
59.39	−0.09	0.96	0.11	136.08	0.15	136.25	19	6
59.07	0.87	−0.09	0.11	134.61	0.15	135.74	19	6
62.19	0.78	−0.66	0.11	149.21	0.15	150.25	19	6
64.98	0.12	−0.55	0.11	162.90	0.15	163.28	19	6

**Table 3:** Kinematic and statistical indicators by mode of transport.

Type	Maximum_Speed (km/h)	Average Speed (km/h)	Minimum_Speed (km/h)	Standard_Deviation (km/h)	RMS (km/h)	Maximum_Acceleration (m/s^2^)	Minimum_Acceleration (m/s^2^)	Average_Acceleration (m/s^2^)	Direction_Changes	Stop_Threshold (km/h)	Stop_Time (s)
Walking	5.68	1.76	0.00	1.83	1.83	1.41	−1.13	−3.42 × 10^−18^	96.00	−1.90	18.00
Bike	50.18	12.13	0.00	10.63	10.63	4.99	−6.45	9.51 × 10^−5^	1977.00	−9.13	92.00
Tram	47.37	16.49	0.00	13.01	13.01	5.14	−3.99	−1.59 × 10^−17^	382.00	−9.53	17.00
Bus	78.34	14.20	0.00	14.75	14.75	3.31	−5.02	−1.30 × 10^−3^	1071.00	−15.30	114.00
Taxi	114.34	21.39	0.00	19.35	19.35	5.21	−6.12	1.44 × 10^−4^	11,854.00	−17.31	636.00
Vehicle	99.34	7.94	0.00	18.30	18.30	10.82	−6.85	−1.50 × 10^−6^	7588.00	−28.66	1074.00

**Table 4:** Validation confusion matrix.

True Class	1	2	3	4	5	6
1	777	1	1	1	115	0
2	2	1662	0	0	32	0
3	0	0	892	0	442	0
4	0	0	0	2912	1923	10
5	9	0	109	470	52,209	17
6	0	1	0	220	341	2875

**Table 5:** Test confusion matrix.

True Class	1	2	3	4	5	6
1	822	2	0	1	70	0
2	2	1663	0	0	32	0
3	0	0	941	0	403	0
4	0	0	0	2901	2095	0
5	8	0	107	216	52,458	17
6	0	1	0	26	315	2860

## Data Availability

The original contributions presented in the study are included in the article, further inquiries can be directed to the corresponding author.

## Abbreviations

Af	Front area
Alt	Altitude
ANN	Artificial neural network
*a_x_*	Longitudinal acceleration
C_X_	Drag coefficient
ρ	Density
Fa	Aerodynamic resistance
Fg	Gravitational resistance
Fr	Rolling resistance force
Fslope	Force slope
GPS	Global position system
Lat	Latitude
Lon	Longitude
m.a.s.l	Meters above sea level
*m*	Mass
OBD	On-board diagnostics
PID	Parameter of identification
RDE	Real driving emissions
ROC	Receiver operating characteristic
